# The multidimensional burden of COVID-19 on Syrian refugees in Lebanon

**DOI:** 10.7189/jogh.11.05003

**Published:** 2021-01-16

**Authors:** Marwan S Hajjar, Ghassan S Abu-Sittah

**Affiliations:** 1 Faculty of Medicine – American University of Beirut, Beirut, Lebanon; 2 Department of Surgery, Division of Plastic Surgery – American University of Beirut Medical Center, Beirut, Lebanon

## Abstract

**Background:**

The COVID-19 pandemic is a global phenomenon that is spreading at an alarmingly high rate, increasing morbidity, mortality as well as affecting the global economy, education sector and psychological well-being of the public. Measures, taken to mitigate the spread of the virus during this pandemic, created challenges to humanitarian communities preventing them from carrying out their responsibilities towards vulnerable populations. The aim of this study is to assess the burden of COVID-19 by looking at the current living conditions, examining available services provided, and identifying the economic and health challenges of Syrian refugee families living in Lebanon.

**Methods:**

This is a cross-sectional study conducted on 129 Syrian refugee families living in Lebanon during the COVID-19 pandemic. All participants provided consent prior to completion of the standardized questionnaire.

**Results:**

During the pandemic, 79% of breadwinners lost their jobs; of those who kept their jobs, 68% had their wages reduced. None of the families was capable of affording all of their basic needs with 55% only partially affording and 45% not able to afford. Thirty percent of Syrian refugee families did not receive support from organizations during the pandemic reflecting the impact of this crisis on humanitarian organizations. Education was also affected as 70% of children did not continue their education at home. Stress and anxiety were the most commonly reported behavioral changes among both children and adults.

**Conclusions:**

The impact of this crisis is multidimensional affecting the economy, global health and education level of the public. Measures should be taken to lessen the detrimental effect of this crisis on the community as a whole and on vulnerable populations in particular.

In the present day, the COVID-19 issue is gaining traction at the public and global health levels, owing to its rapid spread and multifold impact on the physical, mental and psychological well-being of individuals. This phenomenon affected a massive number of individuals leading to devastating outcomes and death. As numbers kept increasing all over the world, the World Health Organization (WHO) declared COVID-19 to be a pandemic on 11 March 2020 [1]. According to recent estimates for mid-September 2020, there are 29 million confirmed cases of COVID-19 with up to 941 000 deaths. Global efforts focus on measures to prevent and mitigate the spread of the virus: this includes wearing masks, frequently washing hands with soap and water as well as adhering to the social distancing protocol, quarantine rules and nationwide lockdown. While the media focuses on the infectivity, rapid spread and critical outcomes associated with the virus, this is only the tip of the iceberg.

First, movement restrictions and activity disruption resulted in a massive hit to the global economy. Studies estimated a sharp decrease in global growth projections that will lead to debts, bankruptcies and financial crisis [2]. Second, in light of the precautions taken, many schools and educational institutions closed worldwide affecting almost 80% of children as per UNESCO estimations [3]. Distant learning through online platforms, media (Television) and paper-based material became essential to maintain continuity of learning [4,5]. However, this course of action is not without issues, as connectivity and accessibility prevented many children, especially vulnerable populations such as refugees, from resuming their education [6]. Third, the psychological impact of this crisis should not be neglected; in fact due to safety measures such as lockdown and quarantine, elevated rates of psychological disorders such as anxiety, stress, depression, harmful alcohol and drug use, even suicide have been reported [7]. Studies show higher rates of anxiety and stress in adults compared to phobias, PTSD and adjustment difficulties in children [8,9]. Fourth, this pandemic is negatively influencing vulnerable communities and families by limiting access to basic needs and services, protection, health care, and education [10,11]. Moreover, public health and containment measures, including nationwide lockdown, raised significant challenges for humanitarian communities worldwide inhibiting them from providing assistance and protection to vulnerable populations [12-15]. As a result, Non-Governmental Organizations (NGOs) and aid programs drastically reduced provision of care and services, in addition, they had to re-adjust the methods and/or type of activities delivered according to the needs identified and assessed.

Today, Lebanon is suffering from an unprecedented series of crises. In October 2019, the Lebanese Economic and Political crises accelerated; in fact, since October, the Lebanese currency lost 78% of its value [16]. The ongoing Lebanese economic crisis and its consequent socioeconomic results, loss of jobs, lack of family support and reduced UN funds; led to worsening living conditions and increased poverty in both refugee and host communities [17-20]. In fact, studies have shown that a considerable number of Syrian refugees live in communities with poor infrastructure and limited resources [21]. Due to poverty, malnutrition and poor infrastructure, these individuals are at an increased risk of infection, health issues and mortality. This was followed by the COVID-19 pandemic and subsequent lockdowns, which exacerbated the situation. To make things worse, on August 4 of 2020, a massive explosion at the city’s port killed hundreds and injured thousands of people leaving many homeless [22]. This unforeseen sequence of events has had a great impact on the Lebanese community as a whole including humanitarian organizations that provide support to refugee families living in the country.

Now, with the Syrian civil war in its ninth year, the socioeconomic status in Syria continues to deteriorate and Syrian families still seek refuge in Lebanon. However due to the current situation in Lebanon, this vulnerable population group keep suffering after displacement as they face several challenges including but not limited to living in hazardous life conditions, lack of adequate access to health care services and threats to their lives with immediate and long-term poor living outcomes.

The aim of this study is to assess the current family conditions and needs during the pandemic, examine available services provided, and identify the economic and health challenges of Syrian refugee families living in Lebanon. This study also aspires to provide additional evidence in order to fill any knowledge gap and allow stakeholders – policy makers, NGOs, international humanitarian organizations and agencies such as United Nations and UNHCR – to better focus their efforts in supporting vulnerable populations during this exceptional multifold crisis in Lebanon.

## METHODS

This study included Syrian refugee families living in Lebanon during the COVID-19 pandemic and Lebanese economic crisis. INARA (International Network for Aid, Relief and Assistance) is an NGO that offers pro bono care for displaced Syrian refugee children with burn injuries [23]. This organization refers these patients to the American University of Beirut Medical Center (AUBMC), a tertiary care center where the authors of this manuscript work. The sample included Syrian refugee families that live in different regions throughout Lebanon and receive care through INARA. INARA caseworkers designed a questionnaire which was pilot tested on staff members belonging to the same community as participants. The questionnaire included measures of family socioeconomic and health status, social and organizational support provided focusing on the child’s psychological and physical well-being during the pandemic, and assessing for online education provision and activities (Appendix S1 in the **Online Supplementary Document**). INARA conducted the data collection through phone calls over the course of nine days in May 2020. INARA caseworkers had the contact information of all participants. Surveys were divided equally among four INARA staff members, who administered the questionnaire by following the same telephone script. All phone calls were conducted in a private setting; in addition, caseworkers de-identified the data collected insuring anonymity. INARA staff members explained the study objectives to participants and took oral consent prior to administering the questionnaire. Although participants were ongoing cases that receive care and supplies from INARA, caseworkers explicitly explained to participants that accepting or refusing participation would not affect the care provided. If subjects accepted to partake in the study, then caseworkers administered the questionnaire. Caseworkers asked participants to respond based on their situation now in comparison to before COVID-19. Most responses were yes/no answers; however, few responses were open ended. The authors of this paper believe there is scientific merit to the data given the immense burden of the COVID-19 pandemic which has left NGOs in a struggle to provide optimal care for this vulnerable population.

The institutional review board (IRB) approved the study protocol and all participants provided informed consent.

### Statistical analysis

The case study sample was comprised of a population size of 139 families, out of which 129 completed the standardized questionnaire. The statistical data analysis was generated in June 2020 using Microsoft Excel (Microsoft Inc, Seattle, WA, USA) spreadsheets. Frequency distributions, descriptive statistics and percentages were carried out for different variables.

## RESULTS

A total of 139 families were contacted, out of which 129 agreed to participate and completed the questionnaire. **Table 1** summarizes the characteristics of the sample. **Table 2** summarizes the economic, social and health implications of COVID-19. Fifty-seven percent of breadwinners were fathers, thirty-eight percent were mothers and four percent were adult sibling of children. Of those, 86% were married, 7% were widowed mothers and 5% were divorced. Majority (77%) of families lived in rented apartment comprised of one or two rooms only, 15% lived in refugee camp and pay rent, 4% in rent-free refugee camp and 4% in residence of friends or family without paying rent. In the sample, 50% of families had six to eight family members living in same household, 27% had nine or more (up-to seventeen) members and 23% had 3 to 5 members living together.

**Table 1:** Descriptive statistics of patient sample (N = 129 families)

	Mean or n	(SD or %)
**Caregiver relation to child:**		
Fathers	74	57.4%
Mothers	49	38%
Adult siblings	5	3.9%
Grandparents	1	0.8%
**Caregiver social status:**		
Married	111	86%
Widowed mothers	9	7%
Divorced	6	4.6%
Single	2	1.6%
Separated	1	0.8%
**Type of residence:**		
Rented apartment comprised of 1-2 rooms only	99	76.7%
Refugee camp with rent	20	15.5%
Refugee camp without paying rent	5	3.9%
Residence of family/friends without rent	5	3.9%
**Number of family members in household:**		
Families with 3-5 members living in same household	29	22.6%
Families with 6-8 members living in same household	65	50.4%
Families with 9 or more members living in same household	35	27.1%

SD – standard deviation

**Table 2:** The economic, social and health implications of COVID-19 in a sample of Syrian refugee families living in Lebanon (N = 129 families)

	Mean or n	(SD or %)
**Income and salary:**		
Caregiver job loss, *yes*	102	79.1%
Caregiver salary cut, *yes*	88	68.2%
Family debt, *yes*	119	92.2%
Family receiving support (help) during COVID-19, *yes*	90	69.8%
**Basic needs and education:**		
Ability to afford basic needs (food, shelter, cloths), *yes*	71	55%
Access to education prior to COVID-19, *yes*	74	57.4%
Continued access to education during COVID-19, *yes*	39	30.2%
**Reasons for discontinuing education at home during COVID-19**		
Schools not providing online education	84	65%
Families cannot afford internet, laptops or smartphone	45	35%
**Lockdown and health status:**		
Families experiencing stress at home during lockdown, *yes*	114	88.4%
Behavioral changes in children during lockdown, *yes*	107	82.9%
**Capacity to afford medication, therapy or medical equipment:**		
Able to afford	2	3.4%
Partially capable to afford	25	43.1%
Unable to afford	31	53.5%

SD – standard deviation

**Figure 1:**
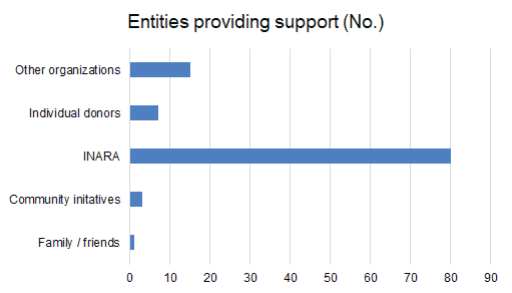
Bar chart showing the distribution of entities that provide support to Syrian refugee families living in Lebanon during COVID-19 (N = 129 families).

Concerning the economic situation, 97 out of 129 families reported that their source of income prior to COVID-19 was wage labor jobs, 65 families received financial support from United Nations, 22 stated that their breadwinners were employed in fixed jobs, 5 received money from charities and another 5 from other family members. Seventy-nine percent of caregivers lost their jobs during COVID-19 pandemic; of those who kept their jobs, 68% had their wages reduced. Some families (70%) received support during COVID-19 vs 30% that did not receive support. Families obtained donations from one or more of the following INARA (80 families), other humanitarian organizations (15 families), individual donors (7 families), community initiatives (3 families) and family/friends (1 family) during COVID-19 crisis (**Figure 1**). None of the families was capable of affording all of their basic needs (food, shelter and cloths) with 55% only partially affording and 45% not able to afford. To note 92% of families had new financial debts during COVID-19.

**Figure 2:**
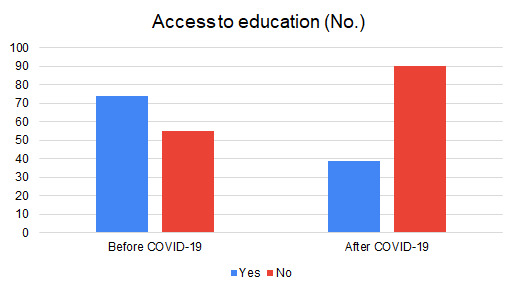
Bar chart showing children access to education before and after COVID-19 in a sample of Syrian refugee families living in Lebanon (N = 129 families).

Regarding the social and educational situation, 85% of families are fully committed to the nationwide lockdown, 13% are partially committed and 2% are not committed. Prior to the lockdown 57% of children were enrolled in schools. There was a worsening in education during the pandemic as 70% of children did not continue their education at home (**Figure 2**). This was explained by lack of online resources provided by schools (65%), lack of internet, laptops or smartphones (35%) (**Table 2**).

In relation to the health status and mental well-being, the majority of families do not have a family member with chronic mental disease (93%), or chronic physical illness (67%) or special needs (85%). In the sample, 54% of families could not afford medication, physiotherapy or medical equipment, 43% could only partially afford vs 3% who could afford it. Self-perceived mental well-being and health was also assessed. A large number of participants (88%) reported constant stress at home due to the lockdown and 83% of children experienced one or more of the following behavioral changes: anxiety (69 families), aggressiveness (38 families), irregular sleep patterns (22 families), hyperactivity (22 families), irregular eating patterns (15 families), 1 bullied others and 1 got bullied.

## DISCUSSION

In light of the economic crisis caused by both COVID-19 and the current situation in Lebanon, our results suggest a worsening in the quality of life, housing conditions, education of children, in addition to loss of jobs and source of income. We found that 45% of participants could not afford basic needs during the crisis with 92% having new financial debts. Moreover, our results suggest that 79% of Syrian refugee families lost their jobs during COVID-19 and 68% had salary cuts (**Table 2**). Our findings were similar to those of another study by the International Labour Organization (ILO) conducted on vulnerable workers in Lebanon, as it showed that 60% of Syrian refugees were permanently laid-off and 31% temporarily laid-off from jobs. Moreover, the ILO estimated the decrease in wages for both Lebanese and Syrian participants to be more than two-thirds what it used to be in the previous year [24,25]. This devastating effect could be explained by the added effect of the lockdown on the deteriorating market, which simply added fuel to the flame. It is also worth mentioning that in view of this decrease in source of income, the majority of our sample (97%) reported difficulty affording medication, physical therapy or medical equipment. Similar findings by Refugees International report suggested that 35% of Syrian refugees living in Jordan lost their jobs due to COVID-19 [25]. Many studies in the literature investigated the negative impact of this pandemic on the global economic growth. In fact, in spite of efforts to alleviate the economic burden, analysts predicted a 5.2% reduction in global GDP in 2020 labelling it the deepest global recession in decades [2]. This recession could be a result of nationwide lockdown that prevent adequate import/export of supplies as well as reduce access and sale of goods leading to a loss in revenue. Reports on the socio-economic burden of COVID-19 suggest an increase in poverty and inequalities at a global scale leading to increase mortality. In fact, Mahler et.al estimated that the pandemic will push 71 million people into extreme poverty earning less than 1.9$ per day in 2020 [26].

In view of the deteriorating economy, humanitarian and aid agencies face challenges in providing care to vulnerable populations. Findings in this study reflect the extent of these implications as 30% of Syrian refugee families did not receive support from organizations during COVID-19. This could be explained by the reduced funding and decreased donations that humanitarian organizations and NGOs are receiving during the pandemic. The Institute of Fundraising (IoF) conducted a survey on 550 charities in the United Kingdom, it estimated a 48% loss of voluntary income and a third of total income of NGOs [14,27]. A different study showed that 98% of Geneva-based NGOs were affected by the COVID-19 pandemic with 79% forced to decrease their activities. Moreover, the survey showed that 42% of Geneva based-NGOs received delayed income from their donors, 36% received reduced income and 14% had their income withdrawn [28,29],

Today, distant learning made it possible to maintain adequate education while adhering to social distancing protocol. This evolution of learning was achieved through digital transformation of education such as e-learning, online platforms and media. However, it is important to note that this technique is far from perfect and leaves many children particularly those belonging to a vulnerable population without education widening inequalities. Our findings suggest that the majority of our sample (70%) did not continue education at home during COVID-19 mainly because of unavailability of online resources and material provided by their schools (65%) or lack of access to the internet, laptops or phones (35%) (**Table 2**). These findings were in accordance with another study conducted in Jordan as 61% of respondents had challenges accessing educational platforms due to slow internet connection (36%) and unavailability of laptops (27%) or phones (12%) [11].

The COVID-19 pandemic is a global phenomenon that is spreading at an alarmingly high rate, increasing morbidity, mortality and affecting the physical, mental and psychological well-being of the public. In fact, our data showed a higher rate of anxiety among adult participants due to the lockdown (88%) as well as increased anxiety, stress, aggressiveness, hyperactivity, irregular sleep and eating patterns, bullying and violent behaviors among children. In addition, the literature reports an increase in substance use, depression and suicidal ideation. One study conducted in the United States showed that 41% of respondents had at least one adverse mental condition, 31% reported anxiety or depression, 11% considered suicide and 13% started using substances to cope with stress and emotions [7]. Moreover, according to a WHO survey, due to the increasing number of infections, many hospitals reassigned personnel to assist with COVID-19 leading to inadequate treatment of non-communicable diseases such as cardiovascular emergencies, hypertension, diabetes and cancer [30]. Furthermore, due to lockdowns and social distancing measures, many non-essential procedures are being postponed causing a deterioration of health care economy that leads to a shortage of staff, lack of medicine and finally bankruptcy [30,31].

In April 2020, the United Nations shed light on the pressing issue of the socio-economic burden caused by the pandemic. The UN issued a framework to help inform and tailor the responses of governments to recover from the crisis, flattening the human suffering curve, bankruptcy and recession curves. This framework is dependent on providing health and social protection to contain the spread and treat the infected. The UN proposed three pillars, which include fiscal and monetary policies, providing financial support for small-medium enterprises and protecting vulnerable populations [32]. Fiscal and monetary policies can be achieved by redistributing and reprioritizing spending, interest loans and by providing investments in health and education. The government can provide grants and loans to self-employed and small-medium enterprises as well as give extensions on payment of utilities and rent in order to maintain jobs and to ensure business continuity. Finally, protecting vulnerable populations by providing financial support, adequate health care and social insurance, while preventing discrimination and violence, plays an essential role in mitigating the impact of COVID-19 crisis [32,33].

This study showed that the population sample is in dire need of support beyond INARA’s mission of health care provision, which prompts the need to collaborate with expert humanitarian actors to implement adequate program interventions. Our results highlight that future studies can benefit from more extended data and detailed assessments of specific needs in order to help guide NGOs and humanitarian organizations in supporting vulnerable populations.

### Limitations

The study’s findings should be interpreted in the light of the following limitations. First, the observed associations are cross-sectional and the level of care prior to COVID-19 could not be assessed. Second, in light of the series of unforeseen events that happened in Lebanon, it would be difficult to predict the extent of COVID-19 implications on humanitarian organizations taking care of Syrian refugees since this effect might be masked by the Lebanese economic crisis, massive protests and events in the country.

## CONCLUSION

The COVID-19 pandemic show no sign of slowing down as the number of confirmed cases and death toll keep increasing on a daily basis. The impact of this crisis is multidimensional affecting the economy, global health and education level of the public. Humanitarian organizations might face challenges in providing care to vulnerable populations such as Syrian Refugees. There is a need to review, refocus and re-program NGO procedures to avoid humanitarian agency closure and to provide proper effective care to the community.

## Additional material

Online Supplementary Document
