# Supplementary material for: The multidimensional burden of COVID-19 on Syrian refugees in Lebanon
Source: J Glob Health. 2021 Jan 16;11:05003. doi: 10.7189/jogh.11.05003 (PMC7897425; doi:10.7189/jogh.11.05003)
Supplement: Online Supplementary Document [file jogh-11-05003-s001.pdf]

Appendix S1

دراسة حول العائلات خلال وباء كورونا

نسعى الى رصد أوضاع اللاجئين السوريين في لبنان وتأثيرات الحجر الصحي الذي أقرّته الحكومة اللبنانية كإجراء وقائي لمنع انتشار وباء كورونا.
نسعى من خلال هذه الدراسة الى جمع معلومات عن الوضع الاقتصادي والاجتماعي والصحي بشقيه النفسي والجسدي للعائلات التي تعمل معها جمعية إنارة.(INARA)

Study on the effect of COVID-19 on Syrian Refugee families.

The aim of this study is to assess the burden of COVID-19 by looking at the current living conditions, examining available services provided, and identifying the economic and health challenges of Syrian refugee families living in Lebanon and receiving care from INARA.

*Required

1- General questions

1.1 Caregiver relation to child * مقدم الرعاية

____

1.2 Social status * الحالة الاجتماعية

____

1.3 Type of residence * نوع المسكن

Mark only one

- ☐ Apartment/residence rental منزل مستأجر
- ☐ Rent-free residence of family/friends مقيم عند أهل أو أصدقاء بدون بدل إيجار
- ☐ Camp without rent مخيم بدون بدل إيجار
- ☐ Camp with rent مخيم مع بدل إيجار

1.4 Number of family members in the household
عدد أفراد العائلة المقيمين في المسكن*

____

2- Economic situation الوضع الاقتصادي

2.1a What is your primary sources of income before covid? *
ما هي مصادر الدخل الأساسية قبل الكورونا

Check all that apply.

☐ Freelance – عمل حر
☐ Employee – موظف
☐ Money transfers from family - تحويلات من العائلة
☐ Charities - جمعيات خيرية
☐ UN card - بطاقة الأمم

2.1b Type of work *
العمل نوع
Check all that apply.

☐ Daily - يومي
☐ Monthly – شهري
☐ Other:______________________________ -

2.2 Did you lose your job due to covid? *
اذا كان رب الاسرة يعمل هل توقف عن العمل بسبب الكورونا؟

Mark only one.

☐ نعم – Yes
☐ لا - No

2.3 Did you receive a salary cut due to covid? *
هل تمّ إقتطاع جزء من راتب رب الاسرة؟

Mark only one.

☐ نعم - Yes
☐ لا - No

2.4a Are you receiving support during covid? *
هل حصلتم على مساعدات مؤخراً خلال فترة الكورونا

Mark only one.

☐ نعم - Yes
☐ لا - No

2.4b if yes, who provided support?
اذا كان نعم، ماهو مصدرها

Check all that apply.

- ☐ INARA جمعية إنارة
- ☐ Other organizations منظمات أخرى
- ☐ Community initiatives مبادرات اجتماعية
- ☐ Individual donors مساعدة فردية
- ☐ Money transfer from family or friends تحويلات من الأهل والأصدقاء

2.5 Are you still able to provide basic needs? *
هل مازلت قادر على تأمين الحاجات الأساسية؟

Mark only one

- ☐ Able-قادر
- ☐ Unable- غير قادر
- ☐ Partially- أحياناً

2.6 How many meals do you have per day? *
عدد الوجبات في اليوم

Mark only one

- ☐ One
- ☐ Two
- ☐ Three
- ☐ Other: ______________________

2.7 Are you in debt due to covid?
هل أصبح لديك ديون خلال فترة الحجر؟

Mark only one.

- ☐ نعم - Yes
- ☐ لا - No

3. Social situation الوضع الاجتماعي

3.1 Are you committed to the lock-down? *
هل انتم ملتزمون بالحجر الصحي؟

Mark only one.
☐ نعم - Yes
☐ لا - No
☐ جزئيا- Partially

3.2 Did your child have access to education before covid?
هل كان يذهب الطفل الى المدرسة قبل الكورونا؟

Mark only one.
☐ نعم - Yes
☐ لا - No

3.3a Is your child continuing education at home during the lockdown? *
هل مازال الطفل يتابع تعليمه في المنزل

Mark only one.
☐ نعم - Yes
☐ لا - No

3.3b if no, why في حال لا، لماذا

3.4 How does your child spend time during the lock-down? *
كيف يقضي الطفل يومه في الحجر؟

Check all that apply.
☐ دراسة - Studying
☐ أنشطة ترفيهية - Playing
☐ أنشطة تعليمية - Educational activities
☐ Other: _____________

4. Health situation الوضع الصحي

4.1a Does anyone in your family have a chronic mental illness? *
هل لدى أحد أفراد العائلة أمراض مزمنة عقلية

Mark only one.
☐ نعم - Yes
☐ لا - No

4.1b Does anyone in your family have a chronic physical illness? *
هل لدى أحد أفراد العائلة أمراض مزمنة جسدية

Mark only one.
☐ نعم - Yes
☐ لا - No

4.2a Does anyone in your family require special needs? *
هل لدى أحد أفراد العائلة إحتياجات خاصة

Mark only one.
☐ نعم - Yes
☐ لا – No

4.2b If yes, can you still afford physiotherapy, medication or equipment?
في حال نعم، هل مازلت قادراً على تأمين تكاليف العلاج أو الأدوية أو العلاج الفيزيائي أو المستلزمات الطبية

Mark only one.
☐ نعم - Yes
☐ لا - No
☐ جزئيا- Partially

4.3 Do you feel stressed at home due to the lockdown?
هل تعانون من توتر العلاقات في المنزل نتيجة الضغط والتواجد الدائم في المنزل؟

Mark only one.
☐ نعم - Yes
☐ لا - No
☐ جزئيا- Partially

4.4a Did you notice any change in behavior of children during the lockdown?
هل هناك تأثيرات نفسية على الاطفال خلال فترة الحجر الصحي

Mark only one.
☐ نعم - Yes
☐ لا – No

4.4b If yes, what are the changes that you noticed?
في حال نعم، ما هو التأثير على الطفل

Check all that apply.

- [ ] Hyperactivity - حركة مفرطة
- [ ] Being bullied - التعرّض للتنمر
- [ ] Bullying others - ممارسة التنمر
- [ ] Aggressiveness - العدائيّة
- [ ] Violence against others - العنف ضد الآخرين
- [ ] Isolation - الإنعزالية
- [ ] Anxiety/stress - القلق/التوتر
- [ ] Irregular sleep patterns - عدم إنتظام النوم
- [ ] Irregular eating patterns - عدم إنتظام الأكل
- [ ] Neglecting study - إهمال الدراسة
